# Bilateral and Multilevel Vertebral Artery Loop Formation Causing Cervical Radiculopathy: A Case Report and Literature Review

**DOI:** 10.7759/cureus.98705

**Published:** 2025-12-08

**Authors:** Xiaoping Wang, Qifei Duan, Youhui Wu

**Affiliations:** 1 Department of Orthopedics, Zhongshan Xiaolan People's Hospital (The Fifth People's Hospital of Zhongshan) Xiaolan Clinical Institute of Shantou University Medical College, Zhongshan, CHN

**Keywords:** anatomical variation, cervical radiculopathy, cervical spine surgery, conservative management, iatrogenic injury prevention, vascular anomaly, vertebral artery loop formation

## Abstract

We present a case of a 60-year-old man with bilateral and multilevel vertebral artery loop formation (VALF) presenting with chronic cervicobrachialgia and dizziness. Magnetic resonance imaging (MRI) revealed bilateral vertebral artery loops at C4-5 compressing the right C5 nerve root, with computed tomography angiography (CTA) identifying an additional loop at the left V1 segment. The patient's symptoms resolved completely with conservative management over a six-month follow-up period. A literature review comparing similar cases over the past two decades reveals successful conservative and surgical management approaches.

## Introduction

The vertebral artery (VA) is anatomically divided into four segments: V1 (preforaminal segment, from the subclavian artery origin to the C6 transverse foramen), V2 (foraminal segment, ascending through the transverse foramina of C6-C2), V3 (atlantic segment, from C2 to the dura mater), and V4 (intradural segment, from the dura to the vertebrobasilar junction) [1]. The V2 segment has become increasingly vulnerable to iatrogenic injury with the rising number of cervical spine surgical procedures, particularly during anterior and posterior subaxial approaches [1]. While VA injury is uncommon, it can lead to devastating consequences, including stroke, permanent neurological deficits, and death, especially when anatomical variations such as VA loop formation (VALF) are present [2].

VALF represents a tortuous course of the VA that may protrude into the neural foramen or spinal canal. The clinical significance of VALF is twofold: first, it can directly compress neural structures causing radiculopathy; second, it dramatically increases the risk of catastrophic vascular injury during cervical spine surgery or transforaminal epidural steroid injections [3]. Previous case reports have documented VA injuries during routine cervical procedures in patients with unrecognised VALF [4].

Bilateral and multilevel presentation of VALF, as in our case, is exceptionally rare and has not been extensively reported in the literature [2,3]. Recent systematic reviews indicate that VA variations occur in approximately 11% of the population (95% CI: 7-15%), with imaging modalities demonstrating superior detection rates compared to cadaveric studies [5]. This case underscores the necessity for heightened awareness among radiologists and spine surgeons, and the importance of systematic evaluation of VA anatomy in cervical spine imaging studies, particularly when common pathologies such as disc herniation and spondylosis do not adequately explain the clinical presentation.

This case report and literature review provide a comprehensive look at both a recent complex VALF case and the cumulative knowledge in this area over the last 20 years, aiming to elucidate best practices and discuss the ongoing evolution of conservative and surgical management techniques with patient outcomes.

## Case presentation

A 60-year-old male patient presented to our spine clinic with a six-month history of chronic cervicobrachialgia and persistent dizziness. He described recurrent pain radiating from the posterior cervical region to the right trapezius and upper arm. The pain was intermittent but progressively worsening, interfering with his daily activities and sleep. His medical history was unremarkable with no previous cervical trauma, surgery, or significant comorbidities. He was a non-smoker with no vascular risk factors.

He had been referred by another institution with a preliminary diagnosis of C5-6 disc herniation based on initial MRI findings, with a recommendation for surgical intervention. On physical examination, the patient demonstrated significant cervical pain and marked restriction of range of motion in all directions, with the most pronounced limitation during right lateral flexion and rotation. The Spurling maneuver on the right side reproduced and exacerbated his radicular symptoms, while passive shoulder abduction provided symptomatic relief-classic signs of cervical nerve root compression [3]. Notably, no motor weakness, sensory deficits, or pathological reflexes were detected on comprehensive neurological examination. Upper motor neuron signs were absent.

Differential diagnoses, investigations, and treatment

Given the planned surgical intervention at the referring institution, we obtained detailed cervical imaging, including repeat MRI and cervicocranial computed tomography angiography (CTA) for surgical planning. Importantly, we noted that the C5-6 disc herniation identified at the referring institution did not correlate with the patient's C5 radiculopathy symptoms-C5-6 disc herniation typically causes C6 radiculopathy, not C5. This anatomical discrepancy prompted our further investigation.

**Figure 1:**
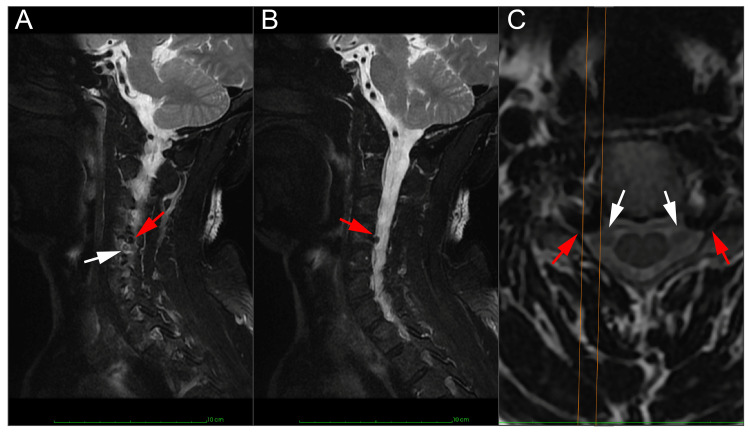
T2-weighted MRI demonstrating bilateral vascular structures at the C4-5 level Bilateral vascular structures (red arrows) at the C4-5 level within the neural foramina, with compression of the right C5 nerve root (white arrows). The bilateral vertebral artery loops are visible as flow voids encroaching into the neural foramina bilaterally.

MRI of the cervical spine revealed bilateral tubular vascular structures at the C4-5 level with medial-posterior coursing loops that had encroached into the bilateral neural foramina (Figure 1). These structures appeared as flow voids characteristic of vascular anatomy, distinct from the typical appearance of disc herniations. The vascular loops were compressing the right C5 nerve root, consistent with the patient's clinical presentation.

**Figure 2:**
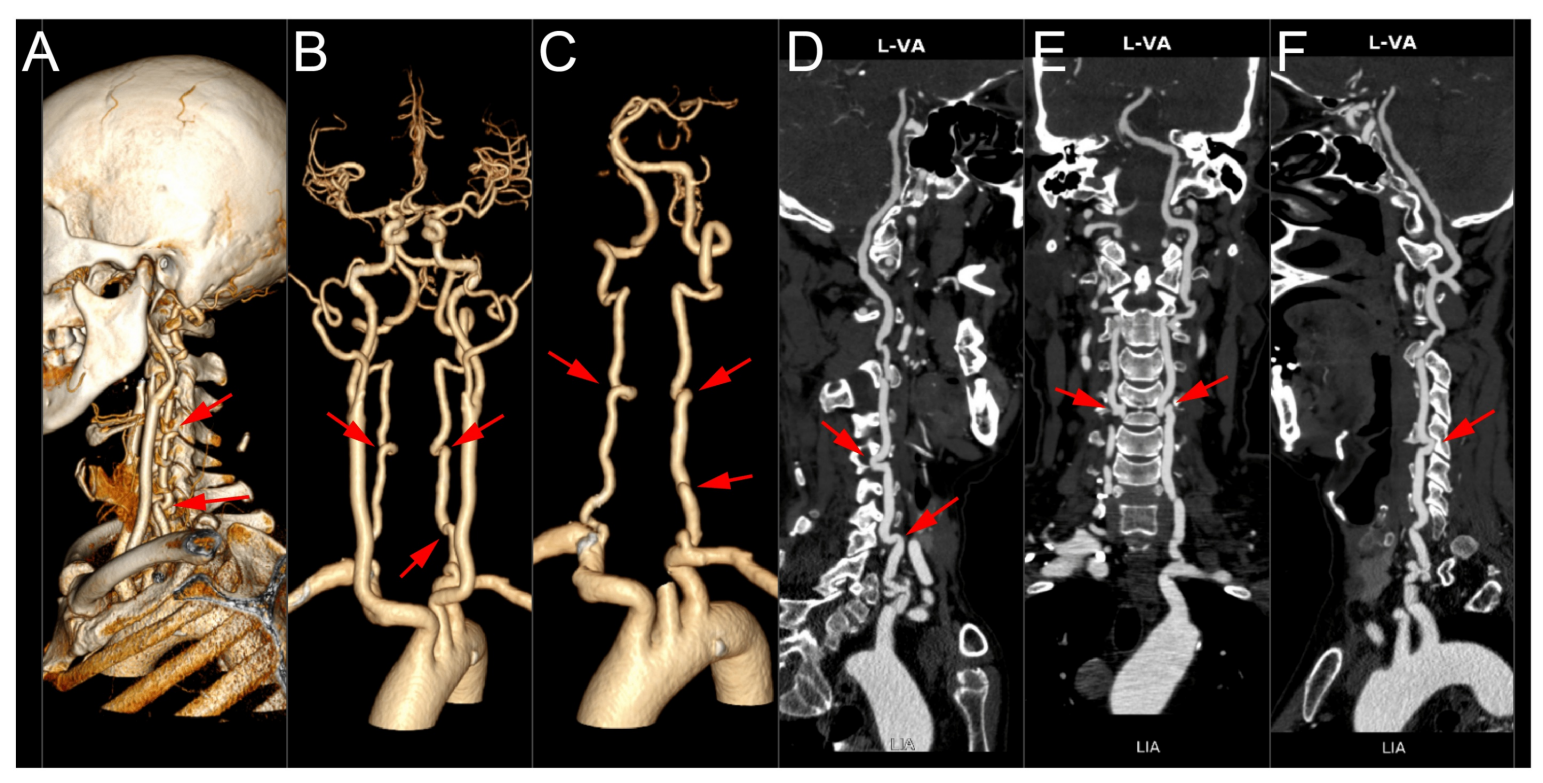
Computed tomography angiography revealing bilateral VALF at the C4-5 level and an additional VALF at the left vertebral artery V1 segment (red arrows) Panels A-F show multiplanar reconstructions demonstrating the tortuous course of the vertebral arteries and their proximity to neural structures. VALF: vertebral artery loop formation

CTA of the cervicocranial vasculature definitively identified these structures as bilateral VALF at the C4-5 level (Figure 2). Additionally, the CTA revealed an unexpected finding: a third VALF at the V1 segment of the left VA. The bilateral C4-5 loops were positioned dangerously close to the planned surgical corridor, and the V1 loop would have been at risk during anterior approaches to the lower cervical spine.

The differential diagnosis included: (i) C5-6 disc herniation with nerve root compression: Initially suspected based on MRI findings and radicular symptoms. However, the level of maximum symptoms (C5 radiculopathy) did not correlate with the C5-6 disc level, which would typically cause C6 radiculopathy; (ii) Cervical spondylotic radiculopathy: Age-appropriate degenerative changes were present but did not adequately explain the pattern of nerve root compression; (iii) VALF: The definitive diagnosis following detailed imaging evaluation. The bilateral vascular loops at C4-5 directly correlated with the C5 radiculopathy, and their position explained both the clinical symptoms and positive provocative maneuvers; and (iv) Vascular causes of dizziness: Given persistent dizziness, vertebrobasilar insufficiency was considered. However, the dizziness was more likely related to cervical proprioceptive input dysfunction rather than vascular insufficiency, as there were no other features of posterior circulation ischaemia [5].

Taking into account the complex vascular anatomy and the significant risk of catastrophic VA injury during surgical intervention, we recommended conservative management as the initial approach. The patient was counselled extensively about the vascular anatomy and the exceptionally high surgical risk associated with any cervical intervention [1,4]. The treatment plan included: cervical collar for intermittent use during symptom exacerbation, physical therapy focusing on cervical stabilisation and postural correction, nonsteroidal anti-inflammatory medications for pain management, and activity modification to avoid positions that exacerbated symptoms

We advised against transforaminal epidural steroid injections due to the risk of arterial injury and subsequent spinal cord infarction [6,7]. The referring institution was contacted and provided with detailed imaging findings, and the surgical recommendation was withdrawn. After extensive discussion, the patient agreed to pursue conservative management.

Clinical course and follow-up

The patient demonstrated remarkable improvement with conservative management. At the six-week follow-up, he reported a significant reduction in cervicobrachialgia with only occasional mild symptoms during prolonged positioning. His dizziness had completely resolved. Physical examination showed improved cervical range of motion and a negative Spurling test.

At the three-month follow-up, the patient was essentially asymptomatic, with no limitations in daily activities. He had returned to his regular exercise routine and was able to perform all occupational tasks without difficulty. The decision to pursue conservative management, rather than surgery, proved to be the appropriate choice given both the clinical outcome and the elimination of surgical vascular injury risk.

At the six-month follow-up, the patient remained symptom-free and expressed satisfaction with the conservative approach. No further imaging or interventions were deemed necessary. He was educated about the permanent anatomical variation and advised to inform any future healthcare providers about the presence of bilateral VALF should cervical procedures be considered [2,8].

## Discussion

A literature review was conducted to examine previous management approaches for VALF and their clinical outcomes in patients with cervical radiculopathy. Electronic databases, including PubMed and Google Scholar, were searched with key terms (vertebral artery loop formation, cervical radiculopathy, anatomical variation, cervical spine surgery, vascular anomaly) relevant to VALF, and their clinical outcomes were used. Articles not presenting data for patients with VALF causing cervical symptoms were excluded. Only articles meeting the criteria of documented VALF with clinical management and outcomes were included. Case reports and case series from all years (2004-2025) were included, yielding a comprehensive review of management approaches spanning two decades. We compared the findings with our case to assess optimal management strategies for this patient population. 

The current case illustrates several critical teaching points regarding VALF in clinical practice. First, VALF can mimic more common causes of cervical radiculopathy, potentially leading to misdiagnosis and inappropriate surgical intervention [2,9]. The initial diagnosis of C5-6 disc herniation would have prompted surgery at an incorrect level and exposed the patient to devastating vascular injury risk.

The bilateral and multilevel presentation in this case is exceptionally rare. While single VALF has been reported in the literature, bilateral involvement at C4-5 combined with an additional loop at the V1 segment represents a unique anatomical variant [2,3]. This underscores the necessity of systematically evaluating the entire VA course when VALF is identified [5].

The pathophysiology of VALF-related radiculopathy involves mechanical compression of nerve roots by the tortuous arterial loop [3]. Dynamic factors may contribute, as arterial pulsations and positional changes can exacerbate compression [10]. This explains the intermittent nature of symptoms and the positive Spurling test in our patient.

From a surgical perspective, unrecognised VALF poses catastrophic risks [1,4]. During anterior cervical approaches, the VA typically lies within the transverse foramina and is protected; however, VALF extends medially beyond this safe zone, placing it directly in the surgical field [1]. Previous reports have documented fatal haemorrhage and stroke from inadvertent VA injury during routine cervical procedures in patients with unrecognised VALF [4]. Recent systematic reviews indicate that VA injury occurs in approximately 0.4% of cervical disc arthroplasty procedures, with surgeon experience being a critical factor (0.33% vs 0.06% for <300 vs >300 cases) [11].

Similarly, transforaminal epidural steroid injections carry substantial risk of arterial puncture and spinal cord infarction when VALF is present [3]. Although the modified approach using specific superior articular process angle measurements has demonstrated zero neurologic or cardiovascular complications in 973 procedures [6], particulate corticosteroids inadvertently injected intra-arterially remain a significant concern [7].

Our case demonstrates that conservative management can be successful for VALF-related radiculopathy, avoiding surgical risks entirely, a finding supported by recent literature showing successful outcomes with integrative approaches [9]. It is important to note that conservative treatment does not alter the underlying vascular anatomy. Rather, symptom resolution likely occurs through several indirect mechanisms: reduction of secondary inflammation around the compressed nerve root, relaxation of reactive cervical paraspinal muscle spasm, neural adaptation to persistent mechanical contact, and optimization of cervical biomechanics to minimize dynamic compression during provocative movements [8,9]. This explains why symptoms can resolve despite the anatomical persistence of VALF.

The etiology of VALF remains incompletely understood. Proposed mechanisms include congenital variations related to embryological development, acquired tortuosity secondary to hypertension and atherosclerosis, and degenerative spondylotic changes causing foraminal narrowing that displaces the artery medially [3,5]. In our patient, the bilateral and multilevel presentation without a significant trauma history may favor a congenital or degenerative origin. However, definitive determination of etiology is not possible in most clinical cases.

When surgical intervention becomes unavoidable, specific techniques have been developed to address VALF safely. The anterior artery release, distraction, and fusion (ARDF) technique achieves indirect loop straightening through neurovascular decompression with sustained pain relief at one-year follow-up [10]. VA transposition via lateral approach with deroofing of the transverse process has also proven effective for multilevel VALF [2].

The imaging evaluation protocol is crucial for preventing surgical disasters. Standard MRI may show vascular loops but can be misinterpreted as disc fragments, tumours, or other pathology [2,8]. When tubular structures are identified in cervical neural foramina on MRI, or when common pathologies such as disc herniation and spondylosis do not adequately explain the clinical symptoms, CTA or magnetic resonance angiography should be obtained for definitive vascular assessment [1,2]. We do not advocate routine CTA for all patients with cervical radiculopathy, but rather a targeted approach based on imaging findings and clinical correlation. Tack et al. documented that the initial plain cervical x-ray showed only widening of the intervertebral foramen, with VALF definitively identified only on subsequent magnetic resonance angiography [8]. Wood et al. reported that loop formations were not radiographically reported on initial MRI in two cases [2].

Radiologists should specifically comment on VA anatomy and clearly communicate anomalies to referring surgeons, while surgeons must actively review imaging for vascular anatomy rather than relying solely on written reports [2,4]. If surgery is unavoidable, risk mitigation strategies include intraoperative navigation, preoperative three-dimensional vascular mapping, and vascular surgery backup [5,11].

Beyond immediate surgical considerations, VA variants are associated with recurrent headaches, vertigo, dizziness, and syncope, suggesting that affected individuals require ongoing monitoring [5]. This reinforces the importance of long-term conservative management and patient education, as exemplified in our patient who remained symptom-free at the six-month follow-up.

## Conclusions

This case demonstrates successful conservative management of bilateral and multilevel VALF, achieving complete symptom resolution at six-month follow-up. The patient's presentation initially mimicked common degenerative cervical conditions, highlighting the diagnostic challenge posed by this anatomical variant. Our experience reinforces the value of obtaining cervicocranial CTA when tubular vascular structures are identified on cervical MRI or when disc herniation and spondylosis do not adequately explain the clinical presentation, rather than as a routine investigation for all patients with cervical radiculopathy.

The favorable outcome achieved through conservative measures in this case suggests that non-operative management should be considered as an initial approach when clinically appropriate, thereby avoiding the substantial vascular risks associated with cervical interventions in patients with VALF. Clear communication between radiologists and surgeons regarding VA anatomy, along with thorough patient education about this permanent anatomical variation, remains essential for optimizing clinical outcomes and ensuring patient safety in future healthcare encounters.
